# Dipyridamole and vascular healing following stent implantation

**DOI:** 10.3389/fcvm.2023.1130304

**Published:** 2023-09-08

**Authors:** Trevor Simard, Richard Jung, Pietro Di Santo, Alisha Labinaz, Spencer Short, Pouya Motazedian, Shan Dhaliwal, Dhruv Sarma, Adil Rasheed, F. Daniel Ramirez, Michael Froeschl, Marino Labinaz, David R. Holmes, Mohamad Alkhouli, Benjamin Hibbert

**Affiliations:** ^1^ Department of Cardiovascular Medicine, Mayo Clinic, Rochester, MN, United States; ^2^ CAPITAL research group, Division of Cardiology, University of Ottawa Heart Institute, Ottawa, ON, Canada; ^3^ Department of Cellular and Molecular Medicine, University of Ottawa, Ottawa, ON, Canada; ^4^ Department of BMI, Faculty of Medicine, Ottawa, ON, Canada

**Keywords:** dipyridamole, neointima, in-stent restenosis (ISR), adenosine, adenosine receptor 2B, optical coherence tomograhy (OCT), vascular smooth muscle (VSMC), proliferation and apoptosis

## Abstract

**Introduction:**

Patients undergoing coronary stent implantation incur a 2% annual rate of adverse events, largely driven by in-stent restenosis (ISR) due to neointimal (NI) tissue proliferation, a process in which smooth muscle cell (SMC) biology may play a central role. Dipyridamole (DP) is an approved therapeutic agent with data supporting improved vascular patency rates. Pre-clinical data supports that DP may enact its vasculoprotective effects via adenosine receptor-A2B (ADOR-A2B). We sought to evaluate the efficacy of DP to mitigate ISR in a pre-clinical rabbit stent model.

**Methods & Results:**

24 New Zealand White Rabbits were divided into two cohorts—non-atherosclerosis and atherosclerosis (*n* = 12/cohort, 6 male and 6 female). Following stent implantation, rabbits were randomized 1:1 to control or oral dipyridamole therapy for 6 weeks followed by optical coherence tomography (OCT) and histology assessment of NI burden and stent strut healing. Compared to control, DP demonstrated a 16.6% relative reduction in NI volume (14.7 ± 0.8% vs. 12.5 ± 0.4%, *p* = 0.03) and a 36.2% relative increase in optimally healed stent struts (37.8 ± 2.8% vs. 54.6 ± 2.5%, *p* < 0.0001). Atherosclerosis demonstrated attenuated effect with no difference in NI burden (15.2 ± 1.0% vs. 16.9 ± 0.8%, *p* = 0.22) and only a 14.2% relative increase in strut healing (68.3 ± 4.1% vs. 78.7 ± 2.5%, *p* = 0.02). DP treated rabbits had a 44.6% (*p* = 0.045) relative reduction in NI SMC content. *In vitro* assessment of DP and coronary artery SMCs yielded dose-dependent reduction in SMC migration and proliferation. Selective small molecule antagonism of ADOR-A2B abrogated the effects of DP on SMC proliferation. DP modulated SMC phenotypic switching with ADOR-A2B siRNA knockdown supporting its role in the observed effects.

**Conclusion:**

Dipyridamole reduces NI proliferation and improves stent healing in a preclinical model of stent implantation with conventional antiplatelets. Atherosclerosis attenuates the observed effect. Clinical trials of DP as an adjunctive agent may be warranted to evaluate for clinical efficacy in stent outcomes.

## GRAPHICAL ABSTRACT

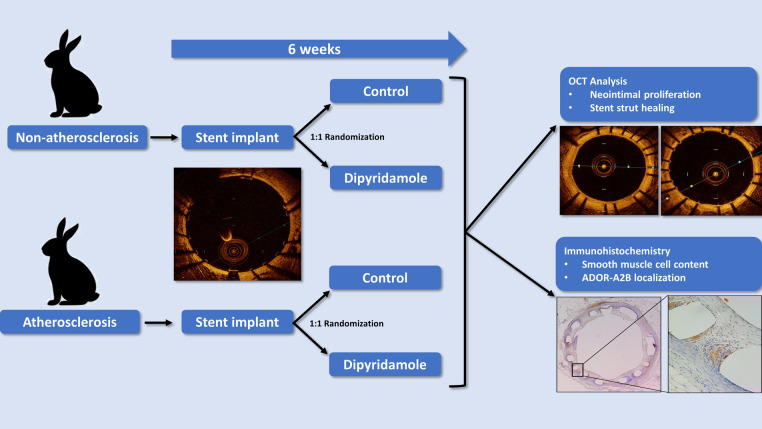
 Non-atherosclerotic and atherosclerotic rabbits were randomized to control or dipyridamole for 6 weeks followed by assessment by intravascular OCT and histology.

## Introduction

Percutaneous coronary intervention (PCI) with stent implantation (1) is still challenged by a 2% annual rate of stent-related adverse events which persist in follow-up (2–4). In-stent restenosis (ISR) due to hyperproliferative neointimal (NI) tissue proliferation, remains a leading cause of stent related adverse events. Despite this, the pathophysiology behind NI proliferation remains incompletely understood, with pathologic smooth muscle cell (SMC) migration and proliferation felt to contribute to some extent (1, 2, 5). ISR remains the focus of ongoing investigation to improve our understanding of the underlying biology and develop novel therapeutics to improve stent-related outcomes (6).

Adenosine (ADO) carries numerous regulatory roles both intracellularly and extracellularly, impacting several cell populations involved in vascular homeostasis, including SMCs (7–9). Adenosine signals primarily via 4 receptors with preclinical work suggesting a potential role for adenosine receptor (ADOR)-A2B in the regulation of the vascular effects observed, particularly NI proliferation (10). Indeed, *in vitro* and *in vivo* arterial injury models supports adenosine mitigating NI formation via ADOR-A2B-mediated inhibition of SMC proliferation (11–13). Despite this promising preclinical work, ADO has yet to establish itself as a viable therapeutic approach for ISR, owing in part to its innate limitations, particularly its short half-life (7–9, 14). However, focused ADOR small molecule agents have demonstrated improved utility and are used broadly clinically (15). Hence, evaluation of alternative agents which modulate adenosine signaling, but with improved clinical pragmatism holds promise for potential therapies.

Dipyridamole (DP) is an established, cost-effective, FDA-approved therapy with a broad range of clinical indications (16). DP improves vascular outcomes in several settings and mitigates restenosis rates following revascularization in both preclinical and clinical studies; however, DP's therapeutic effect in the presence of stent implantation remains largely unknown (17). DP functions primarily as a phosphodiesterase (PDE) 5/6 inhibitor, mitigating cAMP/cGMP breakdown, a mechanism which previously demonstrated clinical benefit for ISR reduction (18). However, DP is also known to augment circulating ADO levels primarily via ENT-1 re-uptake inhibition (7, 17). Preclinical studies suggest that DP may enact its vasculo-protective effects via ADOR-A2B-mediated inhibition of SMC proliferation, though this is based upon *in vitro and* preclinical models with limited data in the context of stent implantation and conventional dual antiplatelet therapy (DAPT) (13).

We have previously reported the utility of a preclinical rabbit stent model for evaluation of stent healing utilizing optical coherence tomography (OCT) (6). Given the hypothesized impact of adenosine biology on vascular remodeling after stenting, we sought to evaluate the efficacy of DP to mitigate ISR in this rabbit stent model, followed by *in vitro* studies to provide mechanistic insights into the observed effects.

## Methods

### Preclinical rabbit model of stent healing

**Figure 1:**
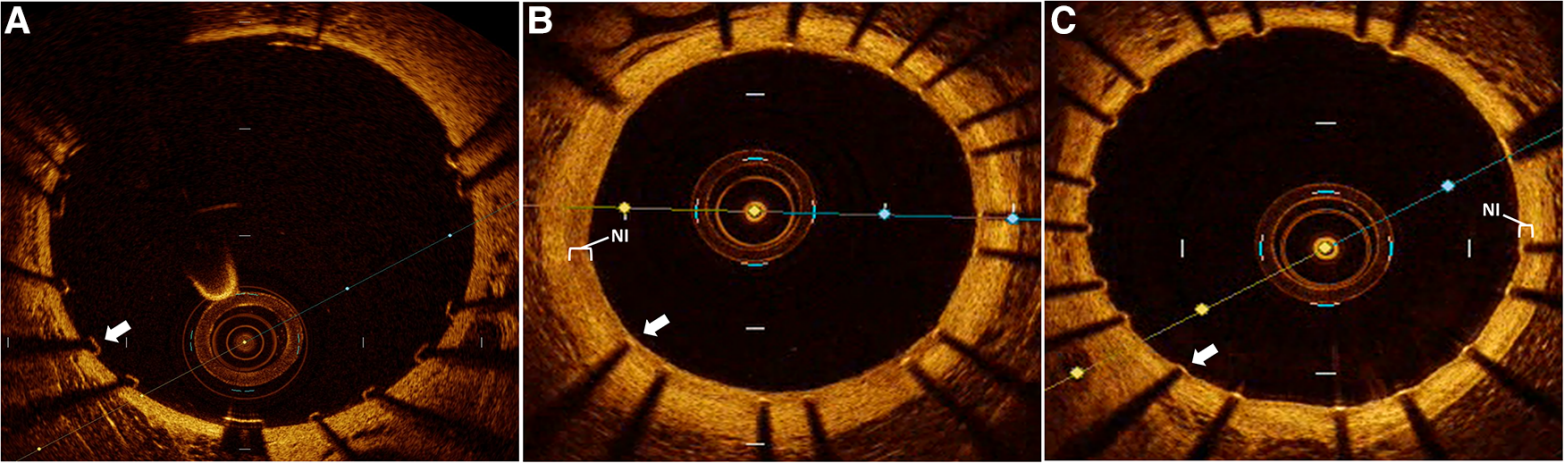
Intravascular optical coherence tomography (OCT) assessment of stent healing. Representative OCT images of rabbit aorta following human coronary stent implantation. (**A**) immediately following stent implantation demonstrating well apposed and completely uncovered stent struts without neointimal (NI) tissue. Followed by 6 weeks post stent implantation with (**B**) control group demonstrating a prominent neointimal (NI, white bracket) burden with all stent struts sub optimally healed due to prominent NI burden overlaying each strut (white arrow), while (**C**) dipyridamole treated subjects demonstrate a minimal NI burden (white bracket) just covering the stent struts with all individual struts optimally healed (white arrow).

**Figure 2:**
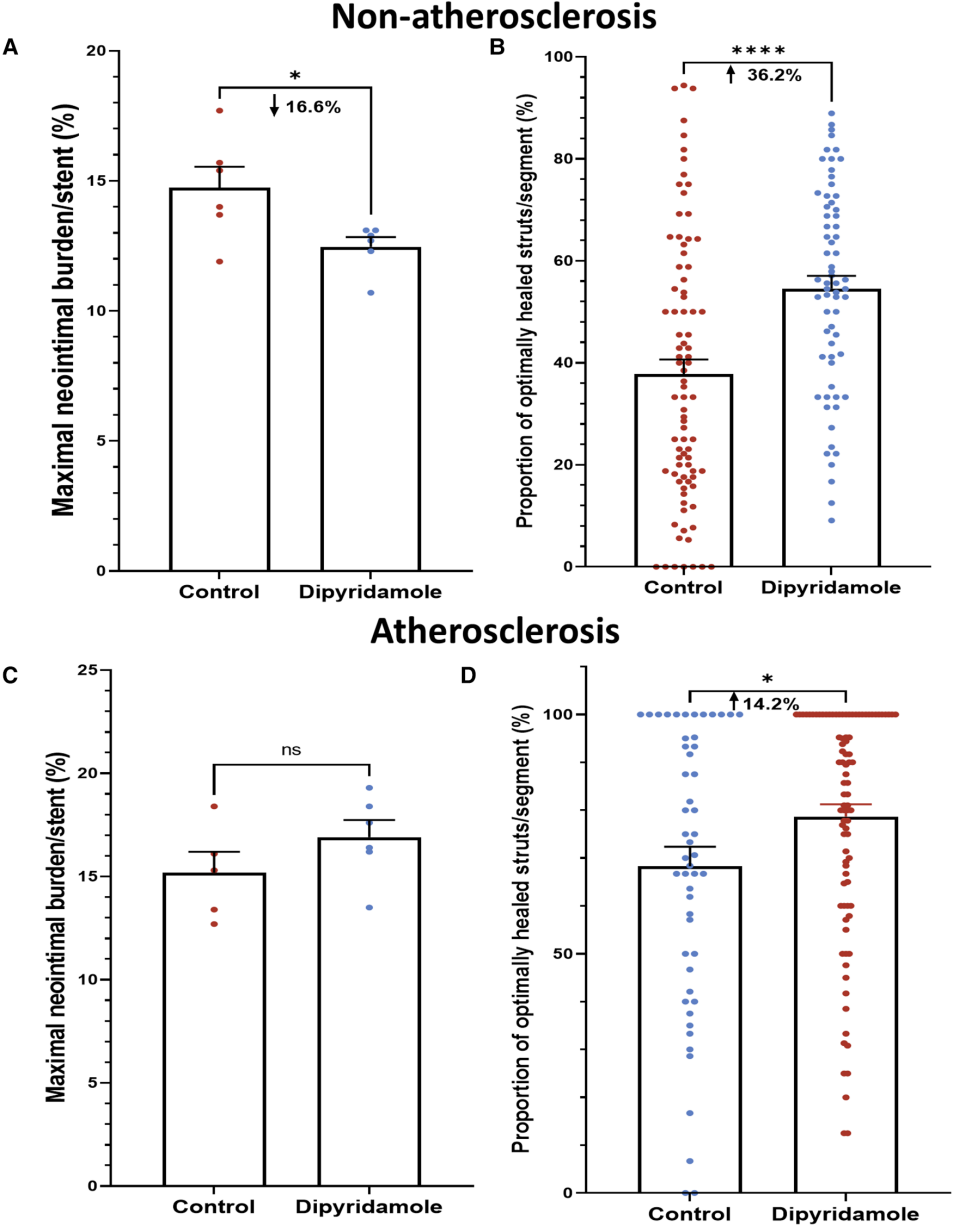
Dipyridamole and stent healing. *Non-atherosclerosis cohort.* Assessment of 12 stents (151 1 mm segments) randomized to control (6 stents, 85 segments) or dipyridamole (6 stents, 66 segments). (**A**) Reduced neointimal burden (%) with dipyridamole assessed by maximal NI volume per stent (16.6% relative reduction, 14.7 ± 0.8% vs. 12.5 ± 0.4%, *p* = 0.03). (**B**) Improved proportion of optimally healed stent struts per segment with dipyridamole (36.2% relative increase, 37.8 ± 2.8% vs 54.6 ± 2.5%, *p* < 0.0001). *Atherosclerosis cohort.* Diet-induced atherosclerosis via administration of 6 weeks of high-cholesterol diet prior to stent implantation. Assessment of 11 stents (143 1 mm segments) randomized to control (5 stents, 51 segments) or dipyridamole (6 stents, 92 segments). (**C**) No difference in maximal NI volume per stent with dipyridamole (15.2 ± 1.0% vs. 16.9 ± 0.8%, *p* = 0.22). (**D**) Improved proportion of optimally healed stent struts per segment with dipyridamole (14.2% relative increase, 68.3 ± 4.1% vs. 78.7 ± 2.5%, *p* = 0.02). Mean ± SEM with comparisons by unpaired Student's *t*-test. Significance denoted as *p < 0.05, **** *p* < 0.0001.

Using our established translational model of stent evaluation in New Zealand White Rabbits, we implanted bare-metal stents designed for human coronaries in the abdominal aortas of equal numbers of male and female rabbits via femoral access, as described previously (6, 19–21). Evaluation of stent implantation in atherosclerotic lesions was performed via addition of 1% cholesterol to the regular diet (Hi Fiber Rabbit Diet, Teklad Envigo, Madison, WI) for 6 weeks prior to stent implantation, as reported previously, with plaques visualized on OCT (6, 22). Following stent implantation all rabbits were maintained on DAPT with ASA (PR 35 mg/2 ml PR gel q2d) and clopidogrel [SQ (ear) 14 mg day prior, then 3.5 mg daily thereafter]. Rabbits (*n* = 12) were then randomized to either control (*n* = 6) or dipyridamole (*n* = 6) therapy for 6 weeks followed by evaluation by both virtual histology with OCT and traditional histology **(Graphical Abstract)**. In the atherosclerosis cohort, 12 separate rabbits underwent stent implantation following induction of atherosclerotic lesions visualized on OCT (23), followed by randomization to either control (*n* = 6) or dipyridamole (*n* = 6) therapy. Dipyridamole was administered daily orally (2.5 mg/kg/day, range 5–7.5 mg/day). Animal studies were approved by the University of Ottawa Animal Care Committee with care provided by the Animal Care and Veterinary Services team. At 6 weeks, virtual histology was performed with intravascular optical coherence tomography (OCT) (Dragonfly Imaging Catheter, Abbott Medical, Minnesota, USA) calibrated per clinical standards, utilizing methodologies previously reported (24–26). Detailed OCT analysis was performed by evaluators blinded to the rabbit sex and treatment group. NI was quantified by measurement of the medial area (MA, mm^2^) and luminal area (LA, mm^2^) combined to report NI quantification as NI% = [MA– LA] / MA × 100% (21). On the vast majority of assessments, the intimal:medial border was readily visualized by OCT and directly measured. However, when this was not well visualized in some areas of a segment, then the outer stent borders were instead measured, and when these were not well visualized then the known stent strut thickness measured from the luminal aspect of the stent strut was used to approximate the location of the intimal:medial border (Figure 1). %NI was quantified in 1 mm segments throughout the stent with the maximal NI area (%)throughout the stented segment, akin to the minimal luminal area(MLA), ultimately utilized, as reported previously (6, 24). The primary endpoint of maximal NI burden per stent was reported as a relative segmental difference on OCT-based quantification to ensure reproducibility (6). Optimal strut healing (OSH) was quantified by OCT as the proportion (%) of OSH struts per 1 mm cross-sectional segment divided by the total number of stent struts present per segment. OSH was defined as the presence of either rhomboid NI tissue on the strut sides with or without minimal luminal strut tissue coverage, while struts that were malapposed, uncovered, or had prominent luminal tissue present were not considered optimally healed, as reported previously (6, 27) (Figure 1). Efficacy of dipyridamole was objectively evaluated in both the non-atherosclerosis and atherosclerosis cohorts via evaluation of the maximal neointimal burden within a stent and the segmental proportion of OSH stent struts, in keeping with prior studies (6). (Figure 2) Plasma adenosine levels were quantified as reported previously (28, 29).

### Immunohistochemistry

At 6 weeks post stent implantation, rabbit aortas were harvested, the stent struts removed and paraffin embedded. They were then deparaffinized, serially washed, incubated in 3% H2O2, washed, blocked for 10 min with 10% goat serum, followed by the respective primary antibody diluted in 1:50 blocking solution and incubated at 4C overnight. Primary antibodies included: SMC (monoclonal, actin antibody, mouse anti-rabbit, MA5-11869, Invitrogen, Carlsbad, CA) and ADORA-2B (polyclonal, goat anti-Human, Novus Biological, Oakville, ON, Canada). Following washing, Vectastain Avidin-Biotin Complex (ABC) kit (Vector Laboratories, PK-6100) was utilized per manufacturer's instruction s (Vector Laboratories, Newark, CA, USA). Biotinylated secondary antibody was then incubated for 10 min at room temperature and washed. Detection was then performed with DAB (3,3′-Diaminobenzidine) solution (Sigma, D5905) and following preparation, 3% H2O2 was added to the solution. DAB solution was then added to the tissue, incubated and washed. Counterstaining with hematoxylin was then performed for 1 min, washed, incubated in PBS for 2 min and then dehydrated. Slides were then mounted with permount solution for examination. Automated quantification of actin antibody signal intensity within the NI tissue was performed and pixels quantified to compare SMC content in control and DP treated cohorts.

### In vitro SMC assessment

(i) *Cell culture and treatments. In vitro* approaches were used to assess the impact of DP on SMC biology as well as focused ADOR-A2B small molecule agents. Treatments included control (dimethyl sulfoxide, DMSO) and escalating doses of DP (10 uM to 200 uM). ADOR-A2B specific small molecule agents included A2B selective agonist (BAY60-6583, 10 uM, Cat #4472, Tocris, Bio-Techne, Minneapolis, MN, USA) and A2B selective antagonist (GS-6201, 1 uM, Cat #4727, Tocris, Bio-Techne, Minneapolis, MN, USA). These treatments were then utilized in varying combinations to delineate the effects of DP and ADOR-A2B-specific factors on SMC biology utilizing our previously described methods (30). Briefly, human coronary artery smooth muscle cells (SMCs, C0175C, ThermoScientific) were maintained at baseline in Medium 231 (M231500, ThermoScientific) with SMC growth supplement (S00725, ThermoScientific) with specific assays as outlined below:
(ii) *Migration analysis.* SMCs were plated onto 96-well plates and incubated until confluence was achieved, labelled with CellMask Orange (C10045, ThermoScientific) and washed. Uniform scratch was then performed with a P200 tip, washed and the appropriate treatments were then added in Medium 231 with serial imaging every 3 h for 24 h with Cytation 5 (BioTek, Winooski, Vermont, USA) and wound closure quantified on the Cytation Gen5 software as percentage confluence.
(iii) *Proliferation and apoptosis analysis.* Cultured SMCs were treated with 5uM CellTrace Violet (C34557, ThermoScientific) and seeded onto 96-well plates (5.0 × 10^3 ^SMC/ml) for 24 h. Following washing, they were then treated with DMSO, DP and ADOR-A2B agonists and antagonists for 48 h at 37°C and 5.0% CO2. Proliferation and apoptosis were then quantified using a MACSQuant Analyzer 10 (software version 2.8.1618.16380, Miltenyi Biotec Inc, Auburn, CA) assessing the CellTrace Violet quantified proliferation (reported as proliferation index ratio to control) and apoptosis via Annexin V-FITC kit (130-092-052, Milteny Biotec) per the instructions for use and reported as % apoptotic cells/total cells present.
(iv) *SMC phenotypic switching—real-time gene expression analysis.* Cultured SMCs on 6-well plates in Medium 231 were treated with DMSO, DP and A2B agonists/antagonists. Total RNA was then extracted with TRIzol LS (10296010, ThermoScientific) and reverse transcription performed with SuperScriptTM IV VILO Master Mix (11766050, ThermoScientific). The cDNA generated was then diluted 1:10 for real-time PCR analysis with SYBR Select Master Mix for CFX (4472942, ThermoScientific) utilizing primers for KLF4, KLF5, SMC actin (ACTA2) and GAPDH to assess SMC phenotypic switching as previously described (30, 31).
(v) *ADOR-A2B Knockdown.* ADOR-A2B knockdown was performed in SMCs in keeping with previously reported approaches in SMCs (11, 32). siRNA analysis was performed utilizing siRNA specific for ADOR-A2B (Catalog # 4390824, ThermoFisher) and silencer selective negative control siRNA (Cat# 4390843, Ambion). These were transfected into SMCs using lipofectamine RNAiMAX reagent (Invitrogen) with successful knockdown of ADOR-A2B confirmed with Western blot analysis for ADOR-A2B 2B (primary antibody, goat anti-Human, Novus Biological, Oakville, ON, Canada) and GAPDH loading control. This approach was replicated with siRNA against ADOR-A2B and negative siRNA control administration followed by treatment with DP 20 uM and A2B agonist and antagonist with subsequent quantification of SMC phenotypic switching as outlined above by RT-PCR analysis of KLF4, KLF5 and ACTA2 gene expression.

### Statistics

We *a priori* calculated the sample size for rabbits based on our prior data suggesting a control % NI proliferation of 15 ± 1.5%, we calculated a relative treatment effect of 15% reduction translating to a treatment group NI volume of 12.75% with an alpha of 0.05 and power 0.9 (beta 0.1) which translated to *n* = 6 per group. Mean ± SEM with comparisons by unpaired Student's t-test and Mann-Whitney where appropriate. Analysis performed with Graphpad Prism 9.4.1 (GraphPhad Software). *P* < 0.05 considered significant with significance denoted as *p < 0.05, ** *p* < 0.01, *** *p* < 0.001, **** p < 0.0001.

## Results

### Dipyridamole and in-stent restenosis

**Figure 3:**
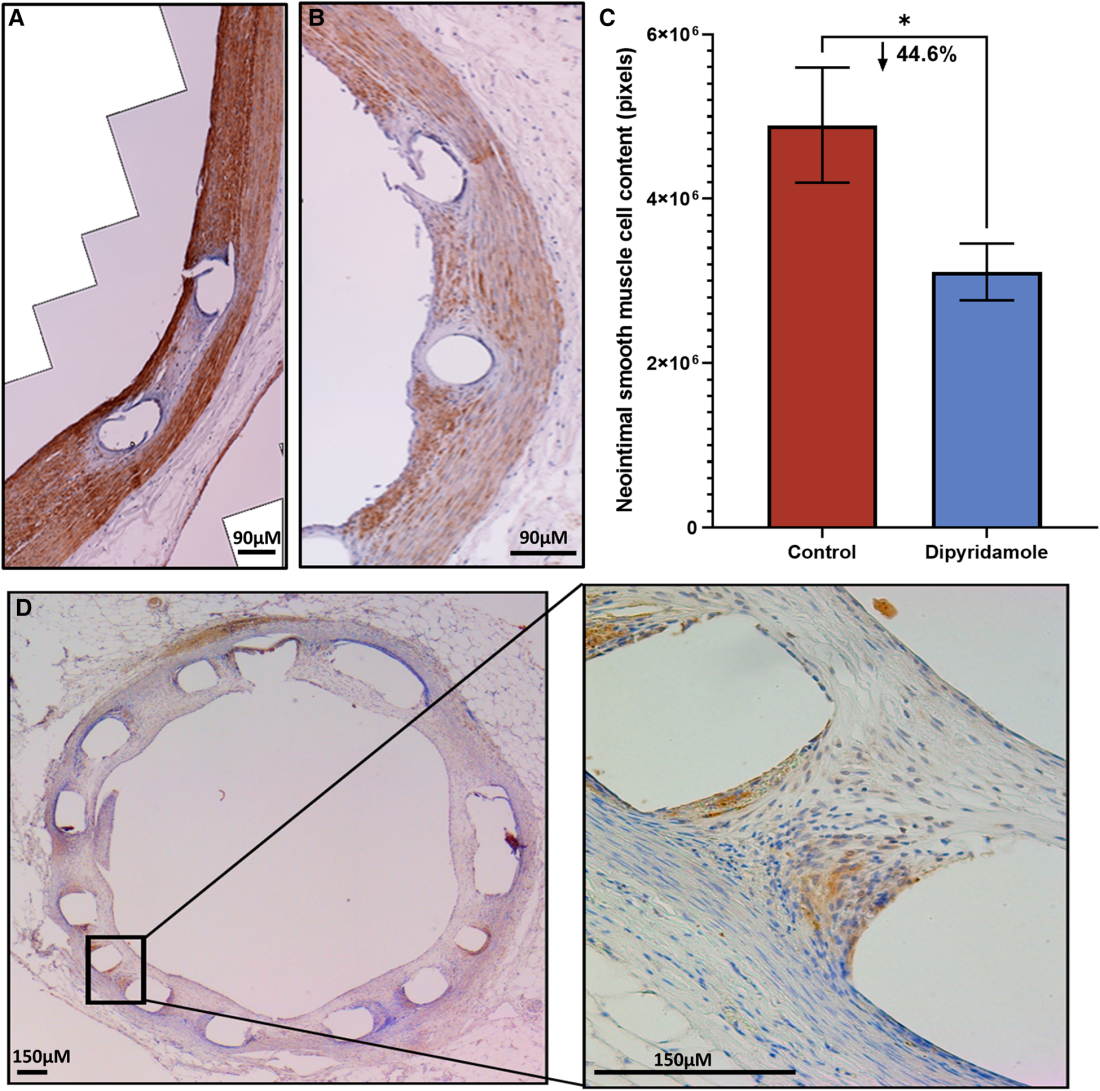
Immunohistochemistry of neointimal tissue. Assessment of neointimal (NI) tissue smooth muscle cell (SMC) content via immunohistochemistry for alpha-SMC actin 6 weeks post stent deployment with either control (3 stents, 2 sections/stent, total 6 sections) or dipyridamole (3 stents, 2 sections/stent, total 6 sections) therapy. Representative immunohistochemistry images of a (**A**) control vessel demonstrating prominent NI tissue with dense SMC content (brown staining) versus (**B**) dipyridamole treated vessel with less prominent NI tissue and SMC content (brown staining). (**C**) Quantification of SMC content by automated detection of signal intensity by pixels with note of 44.6% relative reduction in SMC content with dipyridamole (control 4,895,600 ± 6,995,14 pixels vs. dipyridamole 3,109,225 ± 345,330 pixels, *p* = 0.045). (**D**) Cross-section of rabbit aorta post stent processing with immunohistochemistry for adenosine receptor A2B demonstrating presence of this receptor primarily localized to the peri-strut locations (brown). µM—micrometer. Mean ± SEM with comparisons by unpaired Student's *t*-test. Significance denoted as *p < 0.05.

Utilizing our established preclinical rabbit stent model we performed OCT-guided stent implantation (Figure 1A) followed by intravascular OCT at 6 weeks to assess for stent healing, specifically differences in NI volume and the proportion of optimally healed stent struts between control (Figure 1B) and DP treated cohorts (Figure 1C). In the non-atherosclerosis cohort, 12 rabbits underwent stent implantation (1 stent per rabbit) followed by randomization to control (*N* = 6) or dipyridamole (*n* = 6). Dipyridamole treated rabbits demonstrating a 16.6% relative reduction in maximal %NI/stent (14.7 ± 0.8% vs. 12.5 ± 0.4%, *p* = 0.03) and 36.2% relative increase in the proportion of optimally healed stent struts (37.8 ± 2.8% vs. 54.6 ± 2.5%, *p* < 0.0001) (Figure 2A,B). Next, we assessed the efficacy of DP following stent implantation in the setting of atherosclerosis with 12 rabbits undergoing stent implantation (1 stent per rabbit) followed by randomization (*n* = 6/group) to control or dipyridamole. One rabbit (control group) died prior to the 6 week end point and was unavailable to analyze. In the setting of atherosclerosis, we noted no difference in NI burden between control and dipyridamole (15.2 ± 1.0% vs. 16.9 ± 0.8%, *p* = 0.22) and a 14.2% relative increase in strut healing with dipyridamole (68.3 ± 4.1% vs. 78.7 ± 2.5%, *p* = 0.02) (Figure 2C,D). There was no significant difference in circulating adenosine levels between control and dipyridamole treated rabbits across the entire cohort at baseline, 2,4 and 6 weeks post stent implantation. (Supplementary Figure S1) No overt differences in bleeding diathesis were noted between the cohorts, though hemoglobin levels were not empirically quantified. Immunohistochemistry in the non-atherosclerosis cohort was performed, demonstrating a 44.6% (*p* = 0.045) relative reduction in SMC content in the NI of rabbits treated with dipyridamole over controls with peri-strut localization of ADOR-A2B noted (Figure 3).

### Dipyridamole and the role of ADOR-A2B in SMC migration and proliferation

**Figure 4:**
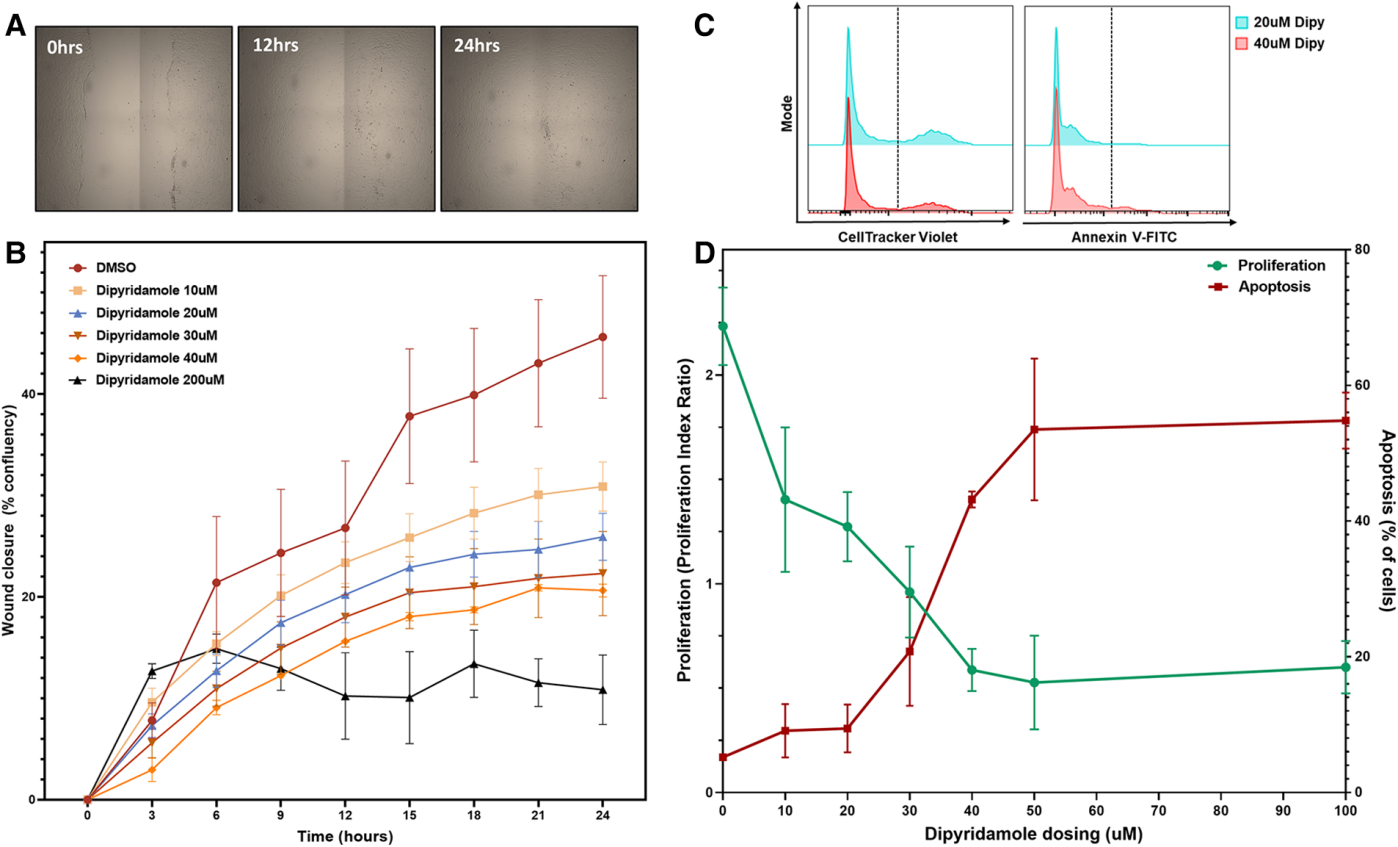
Dipyridamole dose-dependently inhibits coronary SMC migration and proliferation. *In vitro* experiments utilizing human coronary smooth muscle cell (SMC) with treatments as outlined. (**A**) Wound closure assessed by scratch assay on confluent monolayer of SMCs assessed over 24 h with sequential imaging.(**B**) Percentage confluency presented for DMSO control (*n* = 9) and sequential dosing of dipyridamole (*n* = 3/dose) at 10,20,30,40 and 200 uM dosing with diminishing wound closure rates noted with escalating dipyridamole dose compared to DMSO control. At 24 h, wound confluence with DMSO was 40.7 ± 4.7% compared to 25.9 ± 2.3% with 20 uM dipyridamole (44.4% relative reduction, *p* = 0.04). Mean ± SEM with comparisons by Mann-Whitney test. (**C**) Flow cytometry gating for CellTracker Violet (proliferation) and Annexin-V FITC (apoptosis) with representative histograms for 20 uM and 40 uM demonstrating reduced proliferation and increased apoptosis. (**D**) Dose escalation studies with DMSO control (*n* = 3, denoted 0 uM) and sequential escalation of dipyridamole dosing with 10 uM, 20 uM, 30 uM, 40 uM, 50 uM and 100 uM dosing (*n* = 3/dose) demonstrating progressive dose-dependent reduction in proliferation indices (green) with concomitant rise in proportion of apoptotic cells (red). Crossover between 30–40 uM dipyridamole dose and maximal inhibition of proliferation without significant rise in apoptosis noted at 20 uM dipyridamole dosing. Mean ± SEM demonstrated.

**Figure 5:**
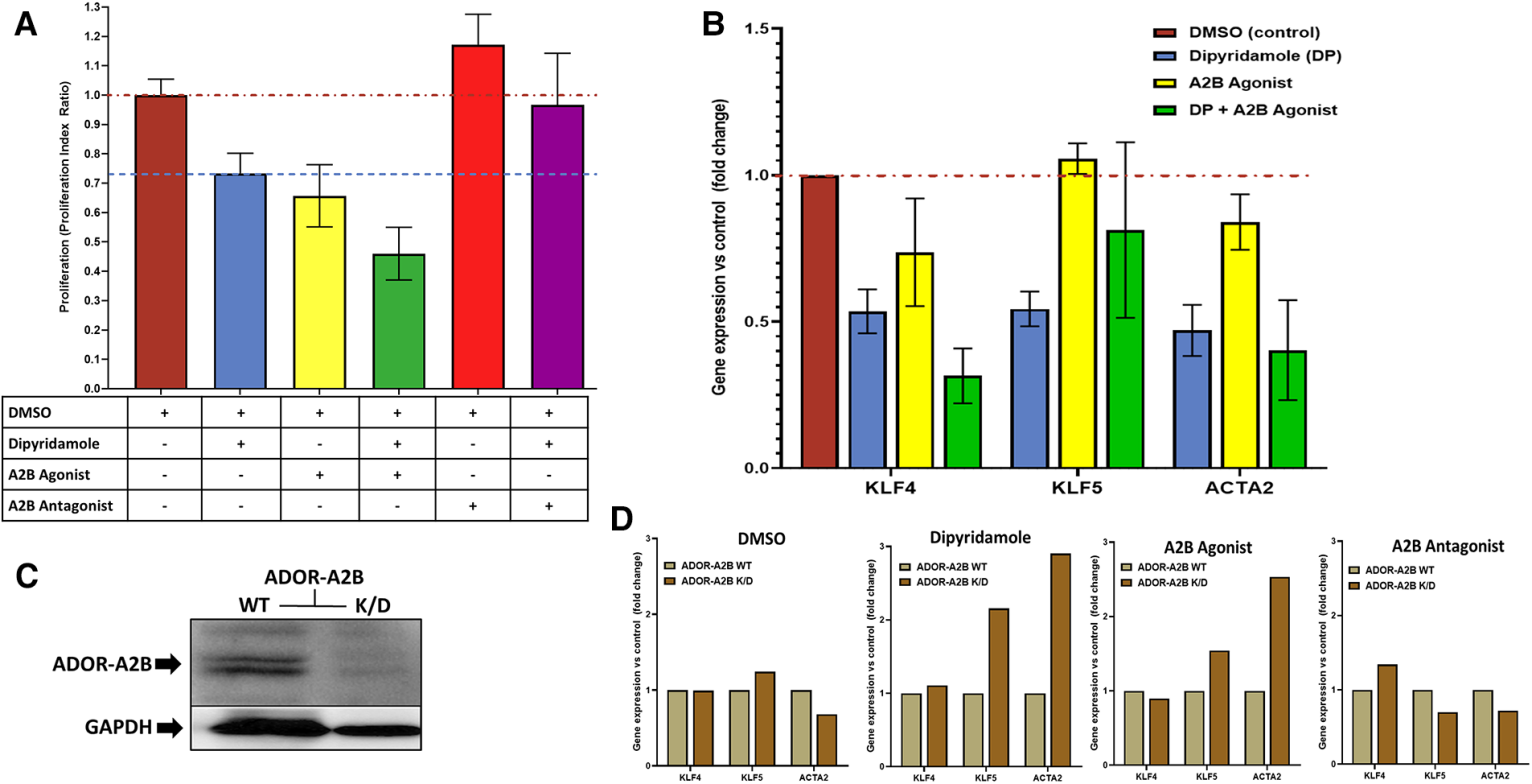
Dipyridamole, ADOR-A2B and the effects on SMC proliferation and phenotypic modulation. (**A**) Flow cytometric assessment of cell proliferation via proliferation index utilized to assess treatment impact on coronary smooth muscle cell proliferation *in vitro* following 48 h of therapy. All treatments indexed to DMSO control (*n* = 22) with red dashed line at unity level designating no difference from control. Dipyridamole (DP) 20 uM (blue, *n* = 20) with 26.7% relative reduction in proliferation index to 0.73 ± 0.07 and blue dashed line designating level of proliferation inhibition achieved with dipyridamole for comparison. A2B agonist (yellow, *n* = 10, BAY60-6583, 10 uM) with 34.3% relative reduction in proliferation to 0.66 ± 0.11. Combination of DP 20 uM and A2B agonist (green, *n* = 10, BAY60-6583, 10 uM) with 54.0% relative reduction in proliferation to 0.66 ± 0.11. A2B antagonist (red, *n* = 10) with 17.2% increase in proliferation to 1.17 ± 0.10. Combination of DP and A2B antagonist (*n* = 10, purple, GS-6201 1 uM) with abrogation of previously noted proliferation inhibition with DP to 3.3% relative reduction in proliferation (proliferation index 0.97 ± 0.18). SMC phenotypic modulation assessment via. RT-PCR evaluation of SMC differentiation markers KLF4, KLF5 and ACTA2 (**B,D**). (**B**) DMSO (*n* = 6), dipyridamole (DP, *n* = 6), A2B agonist (BAY60-6583, *n* = 3), DP + A2B agonist (*n* = 4) assessed with reported fold changes in gene expression relative to DMSO control presented. (**C**) Western blot for ADOR-A2B with GAPDH loading control demonstrating successful knockdown of ADOR-A2B with administration of specific siRNA (K/D) and continued presence of ADOR-A2B with siRNA negative control (WT). (**D**) Assessment of therapies in the context of ADOR-A2B WT and siRNA K/D with RT-PCR for KLF4, KLF5 and ACTA2 with DMSO (*n* = 4) and dipyridamole (*n* = 3), A2B agonist (BAY60-6583, 10 uM) and A2B antagonist (GS-6201 1 uM, *n* = 4). Mean ± SEM presented.

*In vitro* assessment of human coronary SMCs was employed to further evaluate the impact of DP and ADOR-A2B mediated effects. SMC migration was assessed utilizing a scratch assay on confluent monolayer of SMCs (Figure 4A) with escalating doses of DP demonstrating a progressive reduction in SMC migration (Figure 4B). At 24 h, 20 uM DP led to a 44.4% relative reduction in wound closure compared to DMSO control (40.7 ± 4.7% vs. 25.9 ± 2.3%, *p* = 0.04) while 200 uM DP dosing was suggestive of SMC cell death. Next, more granular assessment of proliferation and apoptosis was performed utilizing flow cytometry with escalating DP dosing (Figure 4C). Similarly, this demonstrated a dose-dependent reduction in SMC proliferation with a concomitant augmentation in SMC apoptosis. Assessment of these curves noted that dipyridamole 20 uM dosing led to proliferation inhibition without a concomitant rise in SMC apoptosis, supporting its dosing in subsequent assessments (Figure 4D). *In vitro* assessment of SMCs for proliferation indices demonstrated a 26.7% relative reduction in SMC proliferation with DP 20 uM over DMSO control, while addition of DP and A2B agonist (10 uM BAY60-6583) led to a 54.0% relative reduction in SMC proliferation over control. Administration of DP and an A2B antagonist (1 uM GS-6201) abrogated the effect of DP monotherapy with no relative reduction in proliferation noted (Figure 5A). Collectively, this supports that the inhibition of SMC proliferation may, at least in part, be mediated by ADOR-A2B.

### Dipyridamole and the role of ADOR-A2B in SMC phenotypic switching

*In vitro* assessment of SMCs was further utilized to assess modulation of SMC phenotypic switching. Administration of therapies including DMSO control, DP (20 uM), A2B agonist (BAY60-6583 10 uM), DP + A2B agonist with differential modulation of gene expression for KLF4, KLF5 and ACTA2—genes known to characterize differential states of SMC phenotypic switching (Figure 5B). Selective siRNA for ADOR-A2B were transfected into SMCs with confirmed gene knockdown of ADOR-A2B by Western blot assessment (Figure 5C). ADOR-A2B siRNA knockdown was then performed in SMCs prior to treatment with DMSO, DP 20 uM, A2B agonist (BAY60-6583, 10 uM) and A2B antagonist (GS-6201 1 uM) with assessment of gene expression relative to siRNA negative controls demonstrating differential modulation of KLF4, KLF5 and ACTA2 with ADOR-A2B knockdown (Figure 5D).

## Discussion

In-stent restenosis stemming from NI tissue proliferation continues to cause adverse events following percutaneous treatment of obstructive CAD. DP is an established therapeutic agent for improving vascular outcomes with purported adenosine-mediated effects. In a translational stent model with conventional DAPT, we demonstrate that dipyridamole mitigates NI tissue formation and improves stent strut healing, though no difference in NI proliferation was noted in the presence of atherosclerosis. DP mediates these effects, in part, from reduced SMC content driven by diminished SMC proliferation and migration while altering SMC phenotypic switching. Selective modulation of ADOR-A2B and siRNA knockdown supports an ADOR-A2B mediated component to these observed effects. Thus, therapeutic efficacy derived through either reducing NI proliferation or optimizing stent healing may be achievable by either repurposing DP and/or development of ADOR-A2B specific agonists.

Contextualizing our preclinical results within the known clinical data is of importance. Despite advancements and conventional DESs, target lesion failure (TLF) continue to occur at rates of 5.0% in the first year, followed by an annual 2% annual rate up to 5 years which does not plateau—a focus of ongoing study (4). Clinically, DP has had mixed results in reducing restenosis with early PTCA RCTs—not employing a vascular scaffold like a stent—demonstrating no difference in ISR rates (33). Subsequently, RCTs assessing hemodialysis graft patency rates have shown improved graft patency rates from 23% to 28% at 1 year with DP (34). Meta-analyses of all-comer human revascularization studies assessing DP demonstrated a reduction in vascular occlusion rates from 31% without to 23.5% with DP (RR 0.77, 95% CI 0.67–0.88), with a consistent effect across medical regimens and vascular beds (17). However, clinical data assessing DP with conventional stent placement and DAPT remains very limited (35). Hence, the reported 16.6% NI burden relative reduction and improved strut healing in the presence of stent placement is promising. However, the use of BMSs in this study and the abrogated NI reduction with atherosclerosis are important, suggesting attenuated therapeutic effect may be observed in the presence of established atherosclerosis and conventional drug-eluting stents. Nonetheless, given the FDA-approved status of DP, rapid translation to randomized clinical evaluation of DP in addition to DAPT following conventional DES implantation could readily be performed to definitively establish the therapeutic efficacy of DP in the context of real-world atherosclerosis and DES implantation.

Dipyridamole's effect on restenosis and vascular SMCs is well described in preclinical models. Meta-analyses of preclinical models assessing DP therapy demonstrated a 47% relative risk reduction in vascular occlusion post intervention (RR 0.53, 95% CI 0.4–0.7, *p* < 0.00001) in addition to a 13% relative reduction in mean difference of NI burden (standard mean difference −1.13, 95% CI −1.74–−0.53, *p* = 0.0002) (17). Faxon et al. assessed the utility of ASA and DP in rabbit iliac angioplasty model with diet-induced atherosclerotic lesions utilizing histologic assessment at 4 weeks demonstrating an improvement in luminal diameter with oral ASA + DP therapy compared to control (1.3 ± 0.6 mm vs. 0.7 ± 0.6 mm, *p* < 0.05) (36). This effect was felt to be, in part, related to early platelet accumulation with notable thrombosis present in the control group (36, 37). Singh et al. subsequently employed a rabbit model of femoral and carotid balloon injury utilizing local DP delivery (to abrogate concerns of binding to serum proteins systemically), demonstrating a 63% inhibition of SMC proliferation with a 20% reduction in NI thickness (*p* < 0.05) (38). Dubey et al. provided mechanistic insights into these observed DP effects with *in vitro* human aortic SMCs demonstrating inhibition of SMC proliferation and diminished collagen synthesis/extracellular matrix deposition, a plausible means by which DP mitigates NI proliferation (13). However, these studies did not employ conventional antiplatelet regimens nor did they assess stent implantation—important limitations in translating to current day interventional practice. Hence, our study sought to replicate contemporary medical and device therapy finding reduced NI burden, improved strut healing and reduced NI SMC content in the presence of stent placement. Moreover, the dose-dependent inhibition of SMC migration and proliferation observed *in vitro* were robust, with apoptosis noted with escalating doses, but without concern of a cytotoxic/apoptotic effects for the 20 uM dose of DP selected for our *in vitro* experiments. Considering the lessons learned from excessive cellular inhibition with first generation DESs, namely incomplete healing and thrombosis, the appropriate balance of cellular inhibition without toxicity is critical for any stent related therapeutic agent, with dosing varying on whether it is employed systemically or locally (4).

The mechanisms underpinning the purported benefits of DP in mitigating NI proliferation and SMC modulation remain complex, but substantial data supports a connection to adenosine and ADOR-A2B signaling. Preclinical murine wire injury models in ADOR-A2B knockout mice demonstrated augmented NI formation in mice lacking ADOR-A2B (10). Use of 2-chloroadenosine (stable adenosine analog) has been shown to mitigate SMC proliferation, migration and collagen synthesis via activation of ADOR-A2B receptors (11, 13). Dubey et al. documented mechanistic insights centered around cyclin D based on *in vitro* human coronary SMCs with siRNA induced ADOR-A2B knockdown. Briefly, ADOR-A2B signaling leads to activation of adenylyl cyclase leading to augmentation of cAMP and PKA; PKA subsequently inhibits proliferation by blocking several pathways (ERK1/2, Akt and Skp2) that converge at Cyclin D with the collective result being diminished G1 cyclin expression and reduced cell cycle progression as a result (11). This mechanism is also maintained in circulating progenitor SMCs, another documented source of NI proliferation (5, 12). The role of adenosine and circulating progenitors is also supported by rescue of the observed effects by wild type bone marrow transplant in ADOR-A2B knockout mice (10). Hence, ADO is intricately linked to vascular homeostasis and this mechanism demonstrates multiple potentials for DP modulation. DP enacts many effects via PDE 5/6 inhibition which leads to cAMP/cGMP breakdown, hence DP will augment cAMP levels which would stimulate PKA. Indeed, breast malignancy studies have suggested DP induced inhibition of cyclin D1 expression as a mechanism behind diminished malignant cell proliferation (39). Moreover, DP is known to inhibit adenosine re-uptake, thereby augmenting circulating ADO levels from SMC culture *in vitro* (7, 13). Additionally, Dubey et al. suggested that the DP augmentation of ADO levels may mitigate SMC proliferation and collagen synthesis via activation of ADOR-A2B receptors (13). Lastly, altered SMC collagen and extracellular matrix generation suggests another mechanistic insight—phenotypic modulation. Vascular SMCs maintain plasticity allowing substantial variations in phenotype varying from differentiated quiescent forms to de-differentiated forms in response to injury. Numerous signaling pathways modulate this process including SM-alpha-actin (ACTA2) and Kruppel-like factor 4 and 5 (KLF4,5). In response to injury, the de-differentiated “synthetic” phenotype prevails being characterized by a loss of contractile markers and augmentation of proliferation, migration and protein synthesis—if this process persists beyond the initial injury repair, pathologic NI tissue formation ensues (40).

Collectively, our results align with the mechanisms previously reported. We demonstrate that DP has beneficial effects in mitigating SMC content, migration and proliferation without augmenting apoptosis. These beneficial effects are likely mediated via ADOR-A2B-related mechanisms with abrogation of the DP effect noted with selective A2B inhibition. Moreover, DP, selective A2B modulation and A2B siRNA knockdown all result in modulation of SMC phenotypic switching factors—supporting the role of DP and ADOR-A2B in this process. This aligns with prior studies noting DP's impact on diminishing collagen synthesis (13), while also being able to reverse the inhibitory effects of DP via administration of PDGF, supporting a pote ntial role of DP and SMC phenotypic switching (38). While we did not note a clear rela tion between DP administration and elevated circulating adenosine levels, this is not unexpected considering the complex processes involved in circulating adenosine levels (28, 29). Moreover, unchanged systemic adenosine levels do not necessarily preclude tissue-level alterations in adenosine biology which may enact the observed effects.Going forward, clinical evaluation of DP is warranted coupled with ongoing preclinical evaluation of A2B specific agents for mitigating restenosis.

### Limitations

Our study is not without limitations. Our stent model utilizes ideal technical stent implantation parameters with bare-metal stents, enabling refined examination of the biology involved. However, the translatability of these results to real-world clinical practice with less optimal technical stent implants and conventional drug eluting stents may be reduced. Similarly, the atherosclerosis model follows an established approach of diet-induced atherosclerotic lesions, though the plaques developed do not reflect the range of pathologies encountered in human lesions. However, future clinical studies should explore conventional DES implanted in the context of atherosclerosis with complex anatomy where stent and technical stent factors may also contribute to the ISR observed. Nonetheless, these results support further studies in a clinical setting to validate these preclinical findings. Dose selection for DP and empiric selection of 6 week timepoint may not reflect the optimal timepoint for NI tissue assessment with differential kinetics contributing to the results observed. This could be improved with dedicated experiments. The A2B agonists and antagonists employed are highly selective for their specified receptors, though we cannot exclude off target effects on other ADOR's. While the ADOR-A2B siRNA K/D is reassuring as to ADOR-A2B mediated effects, a gene knockout model would be beneficial to fully explore the ADOR-A2B mediated effects.

## Conclusion

Dipyridamole mitigates neointimal formation and improves stent strut healing in a preclinical model of stent implantation with conventional antiplatelet therapy. This therapeutic effect is abrogated in the presence of atherosclerosis. Clinical studies of DP in addition to conventional therapy post stent implantation may be warranted to evaluate for clinical efficacy.

## Data Availability

The raw data supporting the conclusions of this article will be made available by the authors, without undue reservation.
