# Supplementary figures and images for: Dipyridamole and vascular healing following stent implantation
Source: Front Cardiovasc Med. 2023 Sep 8;10:1130304. doi: 10.3389/fcvm.2023.1130304 (PMC10514894; doi:10.3389/fcvm.2023.1130304)

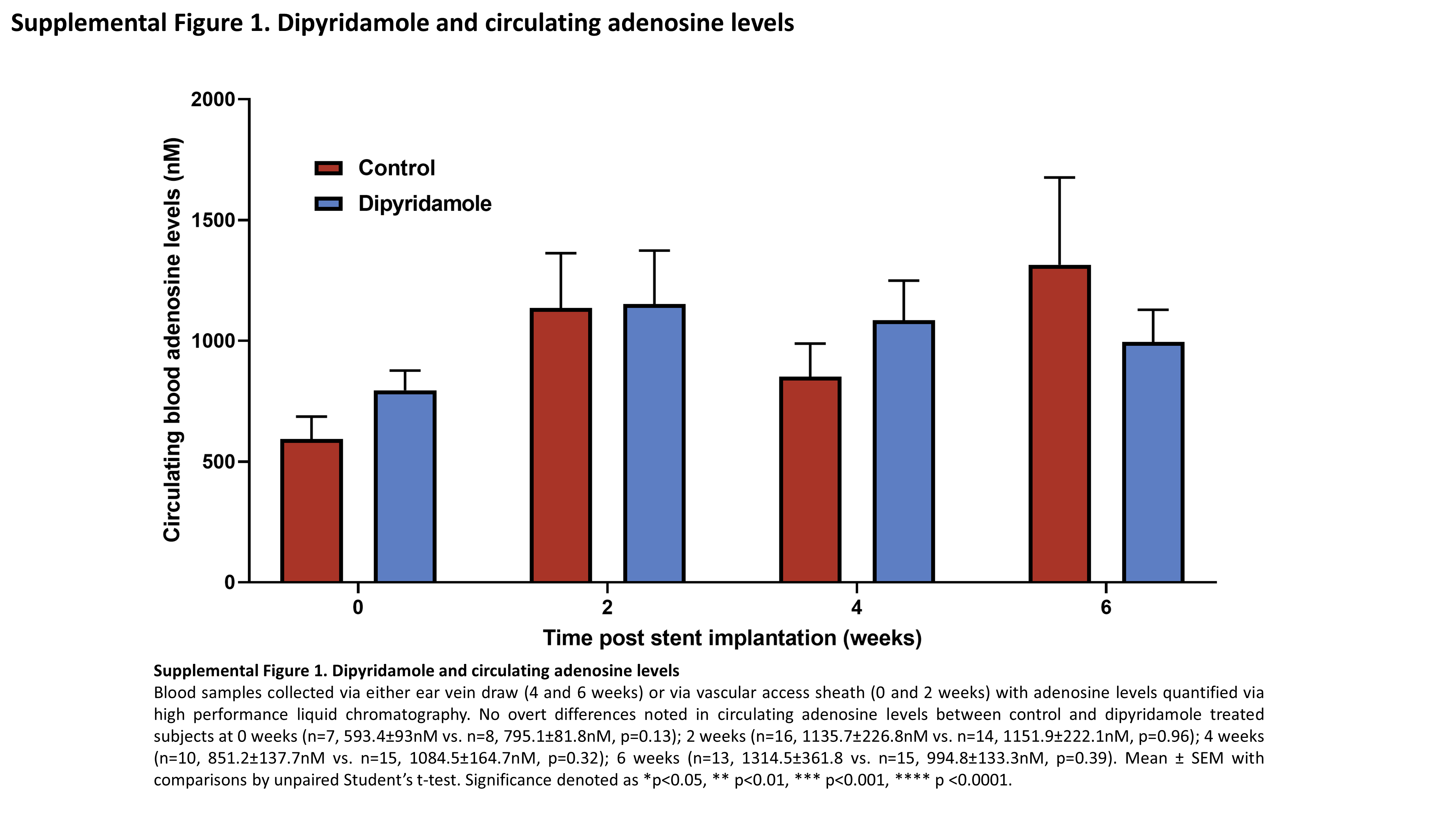

Supplement: Supplementary file 1 [file Image1.tif]
